# Coronavirus pandemic: treatment and future prevention

**DOI:** 10.2217/fmb-2020-0174

**Published:** 2020-11-03

**Authors:** Kenneth Lundstrom

**Affiliations:** 1 PanTherapeutics, Rte de Lavaux 49, 1095 Lutry, Switzerland

**Keywords:** antiviral drugs, clinical trials, COVID-19, pandemic, repurposing, SARS-CoV-2, vaccines

## Abstract

The rapid spread of SARS-CoV-2 leading to the COVID-19 pandemic with more than 400,000 deaths worldwide and the global economy shut down has substantially accelerated the research and development of novel and efficient COVID-19 antiviral drugs and vaccines. In the short term, antiviral and other drugs have been subjected to repurposing against COVID-19 demonstrating some success, but some excessively hasty conclusions drawn from significantly suboptimal clinical evaluations have provided false hope. On the other hand, more than 300 potential therapies and at least 150 vaccine studies are in progress at various stages of preclinical or clinical research. The aim here is to provide a timely update of the development, which, due to the intense activities, moves forward with unprecedented speed.

## Lay abstract

The COVID-19 pandemic took the whole world by surprise by its rapid spread causing enormous destruction to the global health and economy. The unprecedented severity of the disease has led to worldwide death tolls not seen since the Spanish flu pandemic in 1918. The spread of COVID-19 due to modern mobility and the absence of efficient antiviral drugs and vaccines have forced administrations all over the world to place societies under confinements and lockdowns. Accelerated attention has been paid to the development of novel treatments and prevention of COVID-19. The review aims at providing an update of the status on drug and vaccine development to eliminate the pandemic.

Coronaviruses such as the α-coronaviruses HCoV-229E and HCoV-NL63 and β-coronaviruses HCoV-OC43 and HCoV-HKU1 are endemic in human populations and have been associated with 15–30% of annual respiratory tract infections [1,2]. However, the first major human outbreak in 2002–2003 with serious consequences was caused by the SARS-CoV originating in Guangdong in China [3], which resulted in over 8000 recorded cases and 774 deaths [4]. The spread of SARS-CoV was relatively inefficient, which made it controllable through quarantining and helped it to die out in June 2003 [5]. In 2012, another coronavirus-based outbreak occurred in Saudi Arabia and other Middle Eastern countries caused by the novel MERS-CoV [6]. Fortunately, the outbreak did not spread, but still resulted in 855 cases and claimed 333 deaths [7]. Bats have been suggested as the origin of MERS-CoV although dromedary camels can act as intermediate hosts [8], which was confirmed by replication of MERS-CoV in camel cell lines [9] and isolation of an identical virus from a person who had been in contact with an infected camel [10].

SARS-CoV-2 causing COVID-19 was first detected in the city of Wuhan in China in December 2019 and spread quickly throughout the world by person-to-person transmission leading to the worst pandemic since the Spanish flu in 1918 [11,12]. Based on sequence comparison to virus isolated from SARS-CoV-2 infected patients, bats, snakes and pangolins have been suggested as potential carriers of SARS-CoV [12]. One problem with diagnostics and prevention of spread of SARS-CoV-2 relates to its asymptomatic carrier stage [13] and also to the existence of various degrees of severity of COVID-19 ranging from mild flu-like symptoms to pneumonia and death [12]. As there are neither antiviral drugs nor vaccines available, the pandemic has forced countries to take extreme quarantine measurements including closing borders, sealing off hot spot areas, closing down nonessential businesses and air traffic and confining people to their homes. Despite these radical measures more than 37 million people have been tested positive for COVID-19 and at least 1 million deaths have been recorded as of 13 October 2020 [14]. Independent of whether the pandemic will further expand, it will die out or return in a seasonal pattern, there is an urgent need for diagnostics to identify carriers and persons, who have recovered from COVID-19 [15]. Moreover, there is an acute need for developing new drugs against COVID-19. However, efficient vaccines against COVID-19 are absolutely essential for allowing life on the planet to return to what could be considered as normal conditions. In this review, the potential targets for antiviral drugs and vaccine development are described. Moreover, the current situation related to repurposing and novel drugs is summarized. Finally, an update on vaccine development is presented including the current status on preclinical and clinical studies.

## Targets for SARS-CoV

**Figure 1.**
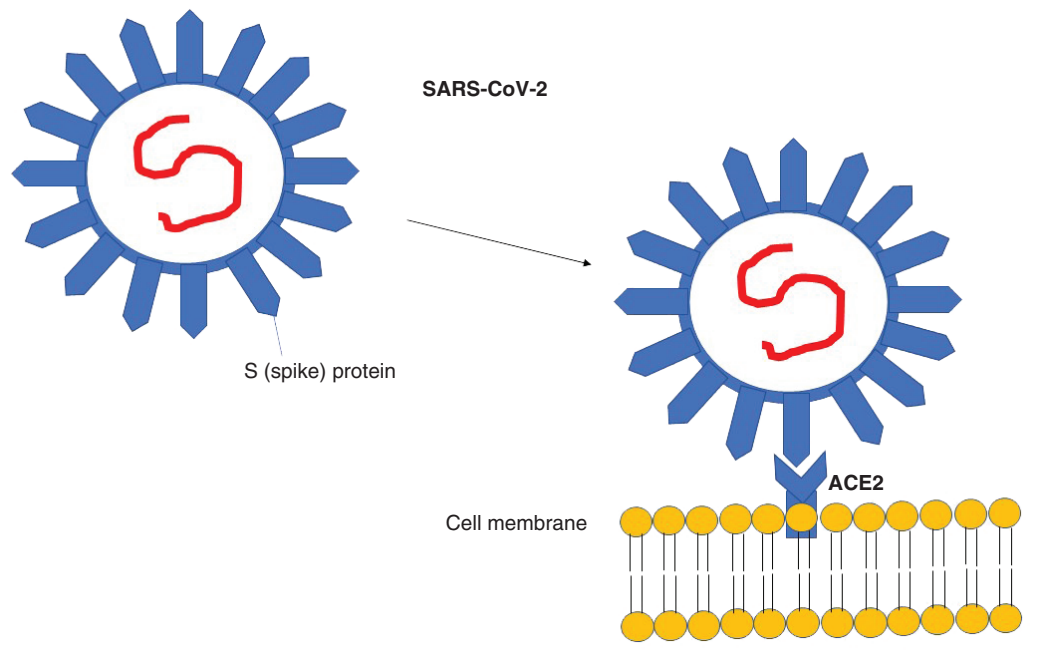
Schematic attachment of SARS-CoV-2 on host cell. The S protein of SARS-CoV-2 attaches to host cells through the ACE2 as the first step of virus entry.

Certain stages of the lifecycle of coronaviruses, particularly the virus attachment and entry (Figure 1), provide potential targets for antiviral drug and vaccine development against SARS-CoV-2 [16]. The ssRNA genome of SARS-CoV-2 is encapsulated by the structural spike S, envelope E, membrane M and nucleocapsid N proteins [17]. The initial attachment of SARS-CoV-2 takes place between the RBD of the S1 region of S the protein and its host cell receptor. This receptor interaction is of great importance as it defines the virus tropism as different coronaviruses target different host cell receptors. For instance, MERS-CoV recognizes DPP4 [18], while both SARS-CoV and SARS-CoV-2 target ACE2 [19]. For this reason, ACE2 has been selected as a drug target for the development of angiotensin receptor blockers, monoclonal antibodies and even plant and mushroom extracts based on traditional Chinese medicine [20]. Replication of viral ssRNA in the cytoplasm presents another important target for antiviral drug development and several drugs acting on RdRp have been demonstrated to decrease viral RNA production [21]. Moreover, RNAi-based gene silencing is another approach to inhibit viral replication [22]. Similarly, vaccine development relies strongly on efficient target identification independent on whether the approach involves subunit or peptide vaccines or vaccines based on viral vector or nucleic acid delivery. The identification of approaches for production of antigens, which can elicit strong neutralizing immune responses and potentially provide protection against challenges with viral pathogens is essential. In the context of drug design, bioinformatics can play an important role. In the case of vaccine development, immuno-informatics can additionally be of great support.

## Repurposing drugs

The urgent need for drugs against COVID-19 has encouraged investigators to verify the potential of repurposing drugs, previously developed as antiviral drugs for other viral or other infectious diseases. For instance, lopinavir-ritonavir has been used for treatment and prevention of HIV/AIDS [23], remdesvir was designed for hepatitis C and Ebola virus disease therapy [24] and hydroxychloroquine as an antimalarial agent [25]. Recently, in search of additional repurposing drug candidates, 26 SARS-CoV-2 proteins expressed in human cells showed protein–protein interaction with 332 human proteins by affinity-purification mass spectrometry [26]. The study further revealed 69 ligands, which included preclinical and clinical compounds as well as US FDA-approved drugs, of which at least five targets and more than ten different chemotypes could present potential antiviral drug targets for COVID-19. Potential repurposing drugs for COVID-19 therapy are summarized and listed in Table 1.

**Table 1.** Repurposing and novel antiviral drugs for COVID-19.

Drug	Disease	Outcome	Ref.
Lopinavir/ritonavir + ribavirin	MERS-like MERS SARS COVID-19 COVID-19	Lower viral load in marmosets with MERS-like disease Clinical trial in MERS patients ongoing Favorable clinical response compared with ribavirin alone No difference to patients receiving standard of care No difference to arbidol treatment	[27] [28] [29] [30] [31]
Faviparavir	COVID-19 COVID-19	Faster viral clearance, improved chest imaging Initiation of Phase III trial in 150 COVID-19 patients in India	[32] [33]
Remdesivir	COVID-19 COVID-19 COVID-19	Clinical improvements in 68% of patients Reduced time to clinical improvement Reduced recovery time of patients	[34] [35] [36]
Hydroxychloroquine	COVID-19 COVID-19	Reduced viral load, but study design and execution suboptimal No advantage of hydroxychloroquine compared with standard of care in COVID-19 patients	[37] [38]
Camostat mesylate	SARS COVID-19 COVID-19	Prevention of SARS-CoV spread in mouse model Block of SARS-CoV-2 entry into lung cells Recruitment in progress for Phase II study in COVID-19 patients	[39] [40] [41]
Sofosbuvir	COVID-19	Planned clinical trial in COVID-19 patients	[42]
Emodin Resveratrol	SARS MERS	Inhibition of SARS-CoV Inhibition of MERS-CoV *in vitro*, potentially also SARS-CoV-2	[43] [44]
Protease inhibitor Ebselen	COVID-19 COVID-19	SARS-CoV-2 inhibition by virtual screening Anti-inflammatory, antioxidant, cytoprotective effects in SARS-CoV-2 infected Vero cells	[45] [46]
ACE2 inhibitor >RBD + Fc	SARS MERS	Efficient inhibition of SARS-CoV in cell cultures Blocking of MERS-CoV infection in mice	[47] [48]
SARS-CoV/CoV-2 mAbs	SARS COVID-19	SARS-CoV mAbs m396 and CR3014 do not bind to SARS-CoV-2 47D11 neutralizes both SARS-CoV and SARS-CoV-2 in cell cultures	[49] [50]
AT1R inhibitors, losartan	COVID-19	Potential drug to be tested in patients with hypertension, diabetic kidney disease for disease outcome/hospitalization	[51]

The combination of lopinavir/ritonavir has been demonstrated to inhibit HIV protease activity and has previously been successfully used for the treatment of HIV/AIDS [52]. Related to coronaviruses, marmosets with a MERS-like disease were subjected to lopinavir/ritonavir treatment, which resulted in improved clinical, radiological and pathological outcomes and reduction in viral load [27]. Furthermore, a multicentre, placebo-controlled, double-blind randomized clinical trial for lopinavir/ritonavir combination therapy is ongoing for hospitalized patients with laboratory-confirmed MERS [28]. Related to SARS, a clinical study in 41 patients treated with lopinavir/ritonavir and ribavirin presented favorable clinical responses compared with ribavirin treatment alone [29].

In the context of COVID-19, 99 patients receiving lopinavir/ritonavir showed no difference in clinical improvement, mortality or detectable viral RNA levels in comparison to control patients subjected to standard of care [30]. In a single-blind, randomized clinical trial, 44 patients with mild or moderate manifestation of COVID-19 received lopinavir/ritonavir or arbidol [31]. In general, no differences were seen for pharyngeal SARS-CoV detection, pyrexia, cough or lung CT scans between the treatment and control groups although the percentage of patients with severe or critical status was higher after lopinavir/ritonavir treatment (38.1%) compared with 12.55 for arbidol and 14.3% for control groups. Based on 143 publications, lopinavir/ritonavir showed no clear benefit compared with standard care [53]. Although there was some reduction in acute distress syndrome, the benefit–risk profile for lopinavir/ritonavir cannot be considered positive for treatment of COVID-19 patients.

The nucleoside analogue favipiravir is a potent inhibitor of viral RNA polymerase showing efficacy against a wide range of influenza viruses [54]. Patients with laboratory-confirmed COVID-19 received either favipiravir or lopinavir/ritonavir in addition to IFN-α [32]. The results demonstrated a shorter viral clearance and a significant improvement in chest imaging for patients treated with favipiravir. Very recently, it was announced that Glenmark Pharmaceuticals will start a Phase III clinical trial in India, where 150 patients with mild to moderate COVID-19 will be enrolled [33].

Much attention has been paid to the use of the adenosine nucleoside triphosphate analogue remdesivir for COVID-19. For instance, compassionate treatment of 53 COVID-19 patients resulted in clinical improvement in 36 patients (68%), discharge of 25 patients and death of seven patients [34]. In another study in China, 158 patients received remdesivir and 79 patients were subjected to placebo, which demonstrated no statistically significant benefits of remdesivir treatment over placebo, although the time was reduced for clinical improvement to occur [35]. Moreover, in a double-blind, randomized, placebo-controlled trial in 1059 adult COVID-19 patients, remdesivir showed shorter patient recovery time (11 days) compared with placebo (15 days) [36]. The Kaplan–Meier estimates of mortality was also lower for patients receiving remdesivir (7.1%) than placebo (11.9%). Next, an additional 5600 patients will be enrolled in the trial, which will be conducted in China, France, Germany, Hong Kong, Italy, Japan, Korea, The Netherlands, Singapore, Spain, Sweden, Switzerland, Taiwan, the UK and the USA.

Chloroquine and hydroxychloroquine with at least 80 registered clinical trials have recently received much attention as potential therapeutics against COVID-19, partly based on ill-founded political over-optimistic statements and on claims from badly designed and executed clinical trials [20]. Although a study on 20 COVID-19 patients in France demonstrated reduction in viral load, the trial design was poor and the results were unreliable with six patients dropping out [37]. In an open label randomized controlled study in 150 hospitalized COVID-19 patients in China, hydroxychloroquine administration did not show any advantage to standard of care treatment alone [38]. In contrast, the study indicated that hydroxychloroquine decreased the survival of hospitalized patients and increased the risk of ventricular arrhythmias. Administration of chloroquine and hydroxychloroquine has also been associated with adverse reactions in patients [55]. For instance, high dose chloroquine caused more severe delayed ventricular repolarisation, QT prolongation, in COVID-19 patients in Brazil [56]. Moreover, patients treated with hydroxychloroquine and azithromycin showed statistically significant changes in QT prolongation suggesting a high risk for arrhythmia [57].

Camostat mesylate, a serine protease inhibitor has been used for pancreatitis and cancer treatment [39]. As camostat mesylate acts as an inhibitor of transmembrane protease serine 2, it was demonstrated to block the spread of SARS-CoV in a mouse model by prevention of the interaction with the CoV S protein [40]. More recently, it was shown that camostat mesylate blocked the entry into lung cells of SARS-CoV-2 isolated from a patient [41,58]. Previously, camostat mesylate has shown very few adverse events and the use of the drug for the treatment of acute symptoms of chronic pancreatitis in more than 100,000 individuals in Japan, only one case of acute eosinophilic pneumonia was reported [59]. Currently, an estimated 114 individuals will be recruited for a double-blind randomized controlled Phase II clinical trial comparing camostat mesylate treatment to placebo in COVID-19 patients [60].

Repurposing sofosbuvir, an anti-HCV antiviral agent, has been based on the high sequence and structural homology of the RdRps of HCV and SARS-CoV-2 [61]. *In silico* modelling suggests that sofosbuvir can tightly bind to the SARS-CoV-2 RdRp leading to viral eradication. Moreover, sofosbuvir is safe, well tolerated and shows high intracellular stability and might therefore be suitable for clinical trials for COVID-19 patients [42].

Finally, as natural products have demonstrated inhibitory effect on viral infection and replication, they might also be adequate for the treatment of COVID-19 [62]. Several flavonoids interfere with activation of the NLRP3 inflammasome showing activity against enteroviruses [63] and Dengue virus [43]. Moreover, the anthraquinone compound emodin produced by many fungi species and found in Chinese herbs has been demonstrated to inhibit the interaction of SARS-CoV S protein with ACE2 [44]. The polyphenol resveratrol, found in high concentrations in grapes, red wine and sprouted peanuts has been shown to inhibit MERS-CoV infection *in vitro* and might also be effective against SARS-CoV-2 [45,64].

## Novel antiviral drugs

In addition to efforts to evaluate antiviral drugs developed for other indications, intense activity in development of novel drugs is in progress (Table 1). In this context, the x-ray structure of the SARS-CoV-2 3CL^pro^ protease alone or complexed with α-ketoamides allowed the design of specific 3CL^pro^ inhibitors with favorable pharmacokinetic properties in mice [46]. In another study, the N3 inhibitor of 3CL^pro^ was identified by combined structure-assisted drug design, virtual drug screening and high-throughput screening computer-aided drug design [65]. The N3 inhibitor demonstrated irreversible inhibition of SARS-CoV-2 3CL^pro^. Additional high-throughput screening identified ebselen, which showed anti-inflammatory, antioxidant and cytoprotective properties in SARS-CoV-2-infected Vero cells.

Obviously for blocking virus entry, ACE2 is a relevant drug target for COVID-19, providing the advantage of targeting the host ACE2 protein and not allowing the virus to circumvent the drug activity by mutations [47]. One approach is to target the small RBD in the S protein, which has been indicated as the key domain for binding ACE2 and it has demonstrated efficient inhibition of entry of SARS-CoV in cell cultures [48]. The same strategy can be applied for the equivalent RBD for SARS-CoV-2. An alternative approach was applied for MERS-CoV, where an Fc fragment was attached to the RBD protein to extend its circulation time and to block viral infection in mice [66]. However, this strategy requires the elimination of cytotoxic Fc domain functions, since the RBD-Fc fusion can also bind to normal cells [67]. A second similar strategy, already demonstrated for SARS-CoV entry and replication [68], relates to the administration of an antibody that binds to the ACE2 protein. Another option is to utilize a nanobody or VHH domains from camelids [69,70]. Importantly, although the ACE2 binding affinity is similar for SARS-CoV and SARS-CoV-2, the furin cleavage site uniquely present in the SARS-CoV-2 S protein provides the means for designing specific SARS-CoV-2 inhibitors [49]. In the context of therapeutic monoclonal antibodies, the first SARS-CoV-2-specific human monoclonal antibody CR3022 demonstrated potent binding to the RBD of SARS-CoV-2 S [50]. However, as CR3022 does not overlap the ACE2 binding site, it might need to be combined with other neutralizing antibodies. Moreover, the need for monoclonal antibodies with specific binding affinity to the SARS-CoV-2 RBD was highlighted by the finding that the potent SARS-CoV-specific m396 and CR3014 neutralizing antibodies did not show binding or the SARS-CoV-2 S protein. Recently, the human 47D11 monoclonal antibody targeting a communal epitope was demonstrated to neutralize both SARS-CoV and SARS-CoV-2 in cell cultures and may potentially offer prevention and treatment of COVID-19 [71].

Another approach, which also could be described as a repurposing drug relates to existing AT1R blockers such as losartan, successfully used for hypertension treatment [51]. AT1R is a valid target as ACE2, which activates AT1R by cleavage of angiotensin I, serves as the binding site for both SARS-CoV and SARS-CoV-2 [72]. A rapid approach would therefore be to investigate whether patients subjected to AT1R antagonist treatment due to hypertension, diabetic kidney disease or other indications have a better disease outcome or a lower frequency of hospitalization than the general population.

Gene silencing based on RNAi has been used for basic research for years [73] but represents a fairly novel approach for treatment of viral diseases [74]. Briefly, RNA molecules can be engineered as siRNAs, shRNAs and miRNAs in the form of 19–23 bp dsRNAs mediating sequence-specific degradation of target mRNA [75]. In the context of coronaviruses, gene silencing has been applied for siRNAs to inhibit SARS-CoV replication in Vero E6 cells (Table 2) [76]. In another study, 48 siRNA sequences covering the SARS-CoV genome were engineered [77]. Transfection of chemically synthesized siRNAs into fetal kidney cells, before or after SARS-CoV infection, resulted in four siRNAs showing potent inhibition of infection and replication. The prophylactic effect of 90% lasted for at least 72 h. Combination of siRNA duplexes also significantly suppressed SARS-like symptoms in rhesus macaques [78].

**Table 2.** RNAi-based gene silencing against coronaviruses.

Delivery	Disease	Effect	Ref.
siRNAs for S1S2/hairpin cDNA siRNAs for S, nsP-12, 13, 16 siRNAs for S, nsP-12 siRNAs for ezrin	SARS SARS SARS SARS	Inhibition of SARS-CoV replication in Vero E6 cells 90% inhibition of SARS-CoV replication in FRhK4 cells Reduced SARS-like symptoms in rhesus macaques Inhibition of actin-binding protein ezrin	[76] [77] [78] [79]
shRNAs for ACE2	SARS	Reduced SARS-CoV infection in Vero cells	[80]
miRNAs for MERS-CoV ORF1ab	MERS	Computational predictions for MERS silencing	[81]
siRNAs for MERS-CoV ORF1ab	MERS	Computational predictions for MERS silencing	[81]
shRNAs for M, N	PDCoV	Decrease in viral titres and RNA levels in ST cells	[82]
shRNAs for M	PEDV	Inhibition of viral RNA replication	[22]
shRNAs for M	SADV	Inhibition of viral RNA replication	[22]
siRNAs for ORF1ab, 3a, s, M, N	COVID-19	Computational design of SARS-CoV-2 siRNAs	[83]

Furthermore, siRNA-silenced ACE2-targeted expression in Vero cells, which also led to reduced SARS-CoV infection [80]. Similarly, siRNA duplexes knocked down expression of the actin-binding protein ezrin, which is known to interact with the SARS-CoV S protein during virus entry [79]. Related to MERS-CoV, four miRNA and five siRNA molecules from the ORF1ab region were rationally designed by computational methods for the silencing of nine MERS-CoV strains [81]. The potency of the *in silico* designed RNAi molecules needs next to be verified for MERS-CoV inhibition in cell lines and *in vivo*. In another study, shRNAs targeting the porcine delta coronavirus (PDCoV) M and N genes showed 13.2- and 32.4-fold reduction, respectively, in titres when swine testicular cells were challenged with PDCoV [82]. Likewise, the viral RNA decreased by 45.8 and 56.1%, respectively. Moreover, shRNAs targeting the M gene of porcine epidemic diarrhea virus (PEDV) and swine acute diarrhea virus and the N gene of PDCoV, expression of each viral RNA was inhibited more than 98% [22]. Additionally, the viral replication was significantly impaired for porcine epidemic diarrhea virus, SADS-CoV and PDCoV. In the case of COVID-19, computational strategies have identified nine siRNAs targeting ORF1ab, ORF3a, S, M and N sequences, which should next be evaluated in cell lines and *in vivo* [83,84].

### Vaccines

The struggle with developing novel or repurposed antiviral drugs against COVID-19 has further strengthened the demand for the need of efficient vaccines against the current SARS-CoV-2, its potential mutated versions and other emerging viruses [20]. Needless to say, there are numerous vaccine development efforts in progress today with more than 100 vaccine candidates at the preclinical stage and at least 13 vaccine candidates in clinical trials [85,86]. The spectrum of approaches is broad comprising live attenuated virus, inactivated virus, protein subunits, nonreplicating and replicating viral vectors and nucleic acid vaccines based on DNA plasmids, mRNA molecules and self-replicating RNA vectors (Table 3). Although numerous studies have been conducted on other animal and human coronavirus vaccines as previously described [20], the focus here is uniquely on COVID-19 vaccines.

**Table 3.** Examples of preclinical vaccine development against COVID-19.

Vaccine/vector	Approach/findings	Affiliation	Ref.
Live-attenuated virus	Generation of multiple SARS-CoV-2 vaccine candidate genomes	Codagenix, The Serum Institute of India	[87]
Inactivated virus	Neutralizing antibodies in rodents, primates, protection against SARS-CoV-2	Beijing Institute of Biological Products Company Ltd, China	[88]
Protein subunit: SARS-CoV-2 molecular clamp	High levels of neutralizing antibodies	University of Queensland, Australia	[89]
Protein subunit: SARS-CoV-2 spherical TMVs	*In vivo* studies in progress	Lomonosov Moscow State University, Russia	[90]
Protein subunit: SARS-CoV-2 baculovirus	Preclinical evaluation in progress	Sanofi-Pasteur, GSK	[91]
Nonreplicating Ad expressing CoV-2 S	Humoral and cellular responses, reduced viral load	University of Oxford, UK, AstraZeneca	[92]
Nonreplicating PIV5 expressing CoV-2 S	Preclinical evaluation in progress	University of Georgia, University of Iowa, IA, USA	[93]
Nonreplicating RABV expressing CoV-2 S	Preclinical evaluation in progress	Bharat Biotech, Thomas Jefferson University, PA, USA	[94]
Nonreplicating MVA expressing CoV-2 S	Preclinical evaluation in progress	GeoVax	[95]
Replicating MV expressing CHIKV VLPs	Preclinical evaluation in progress	Institute Pasteur, Themis, University of Pittsburgh, PA, USA, Merck	[96]
DNA plasmid expressing SARS-CoV-2 S	Antigen-specific T cell responses, Inhibition of SARS-CoV-2	Inovio Pharmaceuticals	[97]
DNA plasmid expressing full-length SARS-CoV-2 S	Protection against SARS-CoV-2 challenges in macaques	Harvard Medical School, Janssen Vaccines	[98]
mRNA-based delivery of SARS-CoV-2 sequences	High levels of neutralizing antibodies after immunization with 2 μg of mRNA	CureVac	[99]
saRNA-based delivery of SARS-CoV-2 sequences	Preclinical evaluation in progress	Imperial College London	[100]

In the context of live-attenuated vaccines, viral deoptimization has been used for the rapid generation of multiple SARS-CoV-2 vaccine candidate genomes for preclinical testing [87]. The vaccine production will then be subjected to scale-up manufacturing and clinical evaluation. Related to inactivated COVID-19 vaccines, the BBIBP-CoV vaccine candidate elicited neutralizing antibodies in mice, rats, guinea pigs, rabbits and nonhuman primates [88]. Vaccination with two doses of 2 μg provided protection against SARS-CoV-2 in rhesus macaques and the good genetic stability for vaccine manufacturing will allow evaluation in clinical trials. Several preclinical studies on protein subunit vaccines are in progress utilizing nanoparticles, virus-like particles, SARS-CoV S spike protein with or without adjuvant, various peptides and molecular clamp technologies [85]. Application of molecular clamp technologies has allowed to lock unstable prefusion versions of surface proteins in a form that shows better immunogenicity [101] and subjected to preclinical studies for the SARS-CoV-2 S protein has demonstrated high levels of neutralizing antibodies against SARS-CoV-2 [89]. In another approach, spherical tobacco mosaic virus particles, previously shown to enhance the immunogenic potential of a rabies vaccine [102], have been applied for COVID-19 vaccine development [90]. Among the expression systems utilized for protein subunit vaccines, baculovirus-based expression of the SARS-CoV S protein has previously induced high titre SARS-CoV-specific neutralizing antibodies in mice [103] and therefore represents a potential alternative approach for vaccine development against COVID-19 [91]. There are numerous preclinical COVID-19 vaccine programs using nonreplicating viral vectors based on adenoviruses, parainfluenza virus, rabies virus and vaccinia virus. For instance, the vaccine based on the adenovirus vector ChAdOx1 expressing the SARS-CoV-2 S protein generated strong humoral and cell-mediated immune responses in mice [92]. Furthermore, a single immunization induced humoral and cellular responses in rhesus macaques and a significantly reduced viral load and absence of pneumonia. In the case of parainfluenza virus-based vaccines, it was shown that a single-dose of intranasal immunization of a parainfluenza virus 5 vector expressing the MERS-CoV S protein induced neutralizing antibody and T-cell responses in mice [93]. Furthermore, a single intranasal administration of 10^4^ recombinant parainfluenza virus 5 particles provided protection against challenges with lethal doses of MERS-CoV. The proof of concept demonstrated for MERS in mice, presents the basis for the development of human vaccines for MERS and COVID-19. In the context of rabies virus, proof of concept has been established for recombinant expression of HIV-1, MERS-CoV, Ebola virus and hepatitis C sequences [94], which makes it a potential vector for vaccine development against COVID-19. Additionally, rabies virus infections target the CNS and the nicotinic acetylcholine receptor in a similar way as has been postulated for SARS-CoV-2, which binds to the nicotine acetylcholine receptor after orthograde or retrograde transport into the CNS. Modified Vaccinia Ankara has been successfully applied for vaccine development against both Ebola virus [104] and Lassa virus [105] and is therefore an attractive candidate for COVID-19 vaccine development. The Modified Vaccinia Ankara-based replication-deficient expression platform has now been mobilized for the preparation of COVID-19 vaccines, currently at the preclinical stage [95]. In another approach, a live-attenuated replication-proficient measles virus was engineered for the expression of CHIKV-like particles, which protected immunized mice from lethal challenges with CHIKV [106] and showed immunogenicity, safety and tolerability in a double-blind, randomized, placebo-controlled Phase II clinical trial [107]. Encouraged by the results from the MV-CHIKV vaccine studies, a preclinical vaccine program was initiated for an MV-based COVID-19 vaccine [96].

Several preclinical vaccine studies using DNA plasmid-based delivery are in progress. For example, a synthetic DNA-based vaccine targeting the SARS-CoV-2 S protein showed robust expression *in vitro* and elicited antigen-specific T cells responses and functional antibodies in immunized mice and guinea pigs [97]. The antibodies neutralized the SARS-CoV-2 infection and blocked S protein binding to the ACE2 receptor. Moreover, a series of DNA vaccine candidates expressing different forms of SARS-CoV-2 S showed humoral and cellular immune responses in rhesus macaques [98]. When challenged with SARS-CoV-2, the full-length S vaccine resulted in significant reduction in viral loads and protection. Similar to DNA-based vaccine development, RNA delivery has also been investigated including nanoparticle and liposome-encapsulated RNA as well as self-replicating RNA vectors [108]. In the case of liposome-encapsulated CHIKV mRNA, intravenous administration provided protection against CHIKV challenges in immunized mice [109], which has paved the way to evaluate mRNA-based approaches for COVID-19 vaccines. In the context of mRNA-based SARS-CoV-2 vaccines, candidates have been selected based on quality criteria and biological activity from preclinical trials [99]. Another approach has been to use self-amplifying RNA for vaccine development against COVID-19 [100].

## Clinical trials

In the context of clinical trials, several trials ranging from Phase I to III are in progress and the most advanced ones are described based on published preliminary results from a Phase I study with an Ad-based vector (Table 4) [110]. In the case of inactivated COVID-19 vaccine produced in Vero cells, healthy volunteers will be subjected to immunization with different doses and exploration of immunogenicity and vaccine persistence in two Phase I/II trials [111,112]. It is anticipated that clinical trials will be completed by the end of the year and the vaccine to reach the market early next year [113]. Another purified inactivated SARS-CoV-2 virus vaccine candidate induced SARS-CoV-2-specific neutralizing antibodies in rodents and nonhuman primates and also provided complete protection in macaques [114] supporting the initiation of clinical trials [115]. A total of 422 subjects, age 60 or older, will be enrolled in a randomized, double-blinded, single-centre, placebo-controlled Phase I/II trial to evaluate the safety and immunogenicity of the vaccine. In a similar study, 774 healthy individuals aged 18–59 years are enrolled [116]. In a Phase I trial on an inactivated SARS-CoV-2 vaccine, 942 healthy volunteers have been enrolled in May 2020 in China [117]. The protein subunit vaccine NVX-CoV2373 is a stable, prefusion protein of the full-length SARS-CoV-2 S applying nanoparticle technology and the saponin-based Matrix™ adjuvant, known for its induction of strong cellular activation of both Th1 and Th2 types, eliciting robust antibody and cytotoxic T-cell responses [118]. The Phase I/II study will be conducted with the SARS-CoV-2 rS nanoparticle vaccine with or without Matrix™ adjuvant in healthy volunteers aged 18–59 years [119,120]. Preliminary immunogenicity and safety results are expected from the Phase I part of the trial shortly and additional data in the randomized, observer-blinded, placebo-controlled Phase II part later.

**Table 4.** Clinical trials for COVID-19 vaccines.

Vaccine/vector	Approach/findings	Stage	Affiliation	Ref.
Inactivated virus	Immunogenicity and vaccine persistence studies	Phase I/II	Wuhan Institute of Biological Products, Sinopharm	[111]
Inactivated virus	Immunogenicity and vaccine persistence studies	Phase I/II	Beijing Institute of Biological Products, Sinopharm	[112]
Inactivated virus + alum	Evaluation of safety and immunogenicity	Phase I/II	Sinovac	[115,116]
Inactivated virus	Study in progress	Phase I	Institute of Medical Biology, Chinese Academy of Medical Sciences	[117]
Protein subunit: full-length CoV-2 S + NP	Study in progress	Phase I/II	Novavax	[119,120]
Nonreplicating Ad expressing CoV-2 S	Study in progress	Phase I/II	University of Oxford, AstraZeneca	[121,122]
Nonreplicating Ad expressing CoV-2 S	Recruitment of patients has started	Phase II/III	University of Oxford, AstraZeneca	[122,123]
Nonreplicating Ad 5 expressing CoV-2 S	Safe, tolerable immunization neutralizing antibody responses	Phase I	Beijing Institute of Biotechnology, CanSino Biological Inc.	[124,125]
Nonreplicating Ad 5 expressing CoV-2 S	Study in progress	Phase II	Beijing Institute of Biotechnology, CanSino Biological Inc.	[126]
DCs transduced LV expressing CoV-2	Study in progress	Phase I	Shenzhen Geno-Immune Medical Institute	[127]
DNA plasmid + electroporation	Study in progress	Phase I	Inovio Pharmaceuticals	[128]
LNP-encapsulated mRNA	Preliminary results of SARS-CoV-2 antibody production	Phase I	Moderna, NIAID	[129–131]
LNP-encapsulated mRNA	Study in progress	Phase I/II	BioNTech, Fosun Pharma, Pfizer	[132]

Several clinical COVID-19 vaccine trials are in progress using Ad vectors. For instance, A Phase I/II study for the ChAdOx1 nCoV-19 vaccine in healthy adults is in progress in the UK to assess the safety, tolerability and reactogenicity of the vaccine [121]. Currently more than 1000 immunizations have been carried out and follow-up is ongoing [122]. Moreover, a Phase III trial for the ChAdOx1 nCoV-19 vaccine, which aims at enrolling up to 10,260 adults and children has started [123]. Moreover, the Ad 5 vector expressing the SARS-CoV-2 S protein was evaluated for safety, tolerability and immunogenicity in a dose-escalation, open-label, nonrandomized, first-in-human trial in China [124,125]. Three doses of 5 × 10^10^, 1 × 10^11^ and 1.5 × 10^11^ viral particles were administered intramuscularly in 108 healthy adults aged 18–60 years. Adverse reactions in the form of injection site pain were mild to moderate and no serious adverse events were recorded by day 28 post vaccination. Neutralizing antibodies were discovered in vaccinees showing a peak humoral response against SARS-CoV-2 at 28 days post vaccination and rapid specific T-cell responses from day 14. More information on the safety and immunogenicity will be received on the Ad-based COVID-19 vaccine from the ongoing Phase II trial [126]. An interesting approach for COVID-19 vaccine development comprise the application of lentivirus transduced dendritic cells (DCs). It has previously been demonstrated that immunization of mice with DCs transduced with lentivirus vectors expressing CD40L and the HIV-1 SL9 epitope induced enhanced antigen-specific T cell proliferation and memory differentiation [133]. In this context, a Phase I trial applying DCs transduced with a lentivirus vector expressing the SARS-CoV-2 S protein (LV-SMENP) has been initiated in China [127].

In the context of nucleic acid-based vaccines, encouraging results from preclinical studies [97] has permitted the initiation of a Phase I clinical trial on a DNA-based SARS-CoV-2 vaccine in 40 healthy volunteers and plans have been made to initiate a Phase II/III follow-up study [128]. Related to RNA-based vaccines, liposome encapsulated mRNA has previously been demonstrated to be efficient for immunization against CHIKV [97] and has also been subjected to vaccine development for COVID-19 [129]. Liposome nanoparticles containing mRNA encoding a prefusion stabilized SARS-CoV-2 S protein was used as the vaccine candidate in a Phase I, open-label, dose-ranging clinical trial to evaluate the safety, reactogenicity and immunogenicity in 150 healthy volunteers [129,130]. Preliminary results from eight participants showed that immunization induced neutralizing antibodies to SARS-CoV-2 at levels for recovered COVID-19 patients for the lower dose of 25 μg while the higher dose of 100 μg elicited significantly higher levels of SARS-CoV-2 antibodies [131]. In addition, the first cohort of healthy adults have been enrolled in a Phase II trial [129]. In total, 300 adult participants aged 18–54 years and another 300 volunteers aged 55 years or older will be enrolled. Moreover, a Phase III study protocol for a randomized, placebo-controlled trial with 30,000 participants has been finalized [129]. Finally, a Phase I/II randomized, placebo-controlled observer-blind, dose-finding trial with four SARS-CoV-2 RNA vaccine candidates has been initiated in healthy volunteers [132]. The safety, tolerability, immunogenicity and potential efficacy at three different dose levels in three age groups, ranging from 18–55, 65–85 and 18–85 years, will be evaluated.

## Conclusion & future perspective

Ultimately, to overcome the COVID-19 pandemic the development of broadly available functional vaccines is of outmost importance and the highest priority [20]. The struggle generally seen with development of efficient antiviral drugs and the necessity of vaccinating the majority of the global population cannot be overstated for regaining the confidence in ‘life returning to normal’. Publicly, the burning questions have been whether an efficient vaccine can be developed and if so, when will it happen? As presented in this review the efforts to successfully develop drugs and vaccines against COVID-19 can be described as unprecedented and have never before reached the size and spectrum of research and development activities bringing together scientists and clinicians from both academic institutions and the pharmaceutical and biotech industry. Although preliminary results from several clinical trials have been encouraging there is no guarantee that the pandemic can be tamed by drugs or vaccines in the near future. Clearly, the accelerated recovery observed for patients treated with remdesivir, the potential of novel drugs targeting ACE2, monoclonal antibodies and RNAi-based gene silencing, although at a relatively early stage of development, are promising. Moreover, the numerous preclinical and the double-digit clinical trials on vaccines have given hope for achieving prophylactic protection for large populations. Obviously, all possible approaches should be considered and therefore the therapeutic potential of plasma from convalescent COVID-19 patients should not be overlooked as it has previously been demonstrated successful for SARS and MERS [134]. In the context of COVID-19, several studies have been conducted [135]. For instance, in a case study, plasma from six donors the anti-SARS-CoV-2 IgM antibody was weakly reactive showing optical density ratios from 1.22 to 2.01 detected by ELISA for all other donors except for one donor with a slightly higher optical density ratio of 5.63 [136]. All donors except one showed high IgG titres (≥1:320), which made them eligible donors. Treatment of a patient with severe COVID-19 allowed release from mechanical ventilation 11 days after the treatment and was then transferred to a general ward.

Although the competition, especially to obtain an efficacious vaccine, is fierce and the spirit is characterized by ‘the winner takes it all’, the impressive diversity of approaches should enhance the probability of success. However, most likely there will be a need for several types of drugs and vaccines due to manufacturing characteristics, the function, target population, longevity and range of activity of drugs or vaccines. In any case, the next 6–12 months will be intense and exciting to follow the drug and vaccine development. It cannot be overstated that for not only medical, but also social and economic reasons we need to overcome the current pandemic and to be better prepared for new waves of COVID-19 and other emerging viruses. It is therefore appropriate to join all efforts to together overcome the COVID-19 pandemic illustrated by the rainbow symbol painted by children all over the world with the message ‘everything will be alright’.

**Executive summary**

**Background**

- The COVID-19 pandemic has caused unprecedented medical, social and economic damage globally highlighting the need for efficient antiviral drugs and vaccines.

**Repurposing drugs**

- A number of studies on repurposed drugs originally developed and/or approved for other viral infections have been subjected to safety and efficacy studies for COVID-19.

**Novel antiviral drugs**

- Novel antiviral drugs targeting viral entry and replication of SARS-coronavirus-2 (SARS-CoV-2) have been designed by computational methods and tested in preclinical animal models.
- RNAi-based gene silencing by nonviral and viral delivery of siRNAs, shRNAs and miRNAs to target viral entry and replication as means of reducing viral loads has been evaluated in cell lines and animal models.

**Vaccines**

- Vaccines based on inactivated and live-attenuated virus, protein subunits, viral vector-based delivery, DNA plasmid and mRNA vaccines haven been verified for immune responses and protection against SARS-CoV-2 challenges in immunized rodents and primates.

**Clinical trials**

- COVID-19 vaccine candidates based on inactivated virus, protein subunits, viral vectors, DNA and mRNA vectors have been subjected to clinical trials in which preliminary results have demonstrated safety, tolerability and immunogenicity.

## References

[1] Bradburne AF, Bynoe ML, Tyrell DAJ Effects of a “new” human respiratory virus in volunteers. Br. Med. J. 3(5568), 767–769 (1967). 6043624 10.1136/bmj.3.5568.767 PMC1843247

[2] Hamre D, Procknow JJ A new virus isolated from the human respiratory tract. Proc. Soc. Exp. Biol. Med. 121(1), 190–193 (1966). 4285768 10.3181/00379727-121-30734

[3] Anderson RM, Fraser C, Ghani AC Epidemiology, transmission dynamics and control of SARS: the 2002–2003 epidemic. Philos. Trans. R Soc. London B Biol. Sci. 359(1447), 1091–1105 (2004). 15306395 10.1098/rstb.2004.1490 PMC1693389

[4] Cherry JD The chronology of the 2002–2003 SARS mini pandemic. Paediatr. Resp. Rev. 5(4), 262–269 (2004). 10.1016/j.prrv.2004.07.009 PMC7106085 15531249

[5] Peiris JS, Yuen KY, Osterhaus AD The severe acute respiratory syndrome. N. Engl. J. Med. 349(25), 2431–2441 (2003). 14681510 10.1056/NEJMra032498

[6] Zaki AM, van Boheemen S, Bestebroer TM Isolation of a novel coronavirus from a man with pneumonia in Saudi Arabia. N. Engl. J. Med. 367(19), 1814–1820 (2012). 23075143 10.1056/NEJMoa1211721

[7] Aleanizy FS, Mohmed N, Alqahtani FY Outbreak of Middle east respiratory syndrome coronavirus in Saudi Arabia: a retrospective study. BMC Infect. Dis. 17(1), 23 (2017). 28056850 10.1186/s12879-016-2137-3 PMC5217314

[8] Meyer B, Muller MA, Corman VM Antibodies against MERS coronavirus in dromedary camels, United Arab Emirates, 2003 and 2013. Emerg. Infect. Dis. 20(4), 552–559 (2014). 24655412 10.3201/eid2004.131746 PMC3966379

[9] Eckerle I, Corman VM, Muller MA Replicative capacity of MERS coronavirus in livestock cell lines. Emerg. Infect. Dis. 20(2), 276–279 (2014). 24457147 10.3201/eid2002.131182 PMC3901466

[10] Azhar EI, El-Kafrawy SA, Farraj SA Evidence for camel-to-human transmission of MERS coronavirus. N. Engl. J. Med. 370(26), 2499–2505 (2014). 24896817 10.1056/NEJMoa1401505

[11] Kaplan EH Containing 2019-nCoV (Wuhan) coronavirus. Health Care Manag. Sci. 23(3), 311–314 (2020). 32146554 10.1007/s10729-020-09504-6 PMC7087552

[12] Yang Y, Peng F, Wang R The deadly coronavirus; The 2003 SARS epidemic and the 2020 novel coronavirus epidemic in China. J. Autoimmun. 109, 102434 (2020). 32143990 10.1016/j.jaut.2020.102434 PMC7126544

[13] Lai CC, Liu YH, Wang CY Asymptomatic carrier state, acute respiratory disease and pneumonia due to severe acute respiratory syndrome coronavirus 2 (SARS-CoV-2): facts and myths. J. Microbiol. Immunol. Infect. 53(3), 404–412 (2020). 32173241 10.1016/j.jmii.2020.02.012 PMC7128959

[14] Worldometer. COVID-19 coronavirus pandemic. (2020). http://www.worldometers.info/coronavirus/

[15] Pascarella G, Strumia A, Piliego C COVID-19 diagnosis and management: a comprehensive review. J. Intern. Med. 288(2), 192–206 (2020). 32348588 10.1111/joim.13091 PMC7267177

[16] Kahn J, McIntosh K History and recent advances in coronavirus discovery. Ped. Infect. Dis. J. 24(Suppl. 11), S223–S227 (2005). 10.1097/01.inf.0000188166.17324.60 16378050

[17] Zhao L, Jha BK, Wu A Antagonism of the interferon-induced OAS-RNase L pathway by murine coronavirus ns2 protein is required for virus replication and liver pathology. Cell Host Microbe 11(6), 607–616 (2012). 22704621 10.1016/j.chom.2012.04.011 PMC3377938

[18] van Doremalen N, Miazgowicz KL, Milne-Price S Host species restriction of Middle East respiratory syndrome coronavirus through its receptor dipeptidyl peptidase 4. J. Virol. 88(16), 9220–9232 (2014). 24899185 10.1128/JVI.00676-14 PMC4136254

[19] Li W, Moore MJ, Vasilieva N Angiotensin-converting enzyme 2 is a functional receptor for the SARS coronavirus. Nature 426(6965), 450–454 (2003). 14647384 10.1038/nature02145 PMC7095016

[20] Lundstrom K Coronavirus pandemic: therapy and vaccines. Biomedicines 8(5), E109 (2020). 32375268 10.3390/biomedicines8050109 PMC7277397

[21] Elfiky AA Ribavirin, remdesivir, sofosbuvir, galidesivir and tenofovir against SARS-CoV-2 RNA dependent RNA polymerase (RdRp): a molecular docking study. Life Sci. 253, 117592 (2020). 32222463 10.1016/j.lfs.2020.117592 PMC7102646

[22] Li K, Li H, Bi Z Significant inhibition of re-emerged and emerging swine enteric coronavirus *in vitro* using the multiple shRNA expression vector. Antiviral Res. 166, 11–18 (2019). 30905822 10.1016/j.antiviral.2019.03.010 PMC7113732

[23] Chandwani A, Shuter J Lopinavir/ritonavir in the treatment of HIV-infection: a review. Ther. Clin. Risk Manag. 4(5), 1023–1033 (2008). 19209283 10.2147/tcrm.s3285 PMC2621403

[24] Warren TK, Jordan R, Lo MK Therapeutic efficacy of the small molecule GS-5734 against Ebola virus in rhesus monkeys. Nature 531(7594), 381–385 (2012). 10.1038/nature17180 PMC5551389 26934220

[25] Ben-Zvi I, Kivity S, Langevitz P Hydroxychloroquine: from malaria to autoimmunity. Clin. Rev. Allergy Immunol. 42(2), 145–153 (2012). 21221847 10.1007/s12016-010-8243-x PMC7091063

[26] Gordon DE, Jang GM, Bouhaddou M A SARS-CoV-2 protein interaction map reveals target for drug repurposing. Nature 583(7816), 459–468 (2020). 32353859 10.1038/s41586-020-2286-9 PMC7431030

[27] Chan JF, Yao Y, Yeung ML Treatment with lopinavir/ritonavir or interferon-β1b improves outcome of MERS-CoV infection in a nonhuman primate model of common marmoset. J. Infect. Dis. 212(12), 1904–1913 (2015). 26198719 10.1093/infdis/jiv392 PMC7107395

[28] Arabi YM, Asiri AY, Assiri AM Treatment of Middle East Respiratory Syndrome with a combination of lopinavir-ritonavir and interferon-β1b (MIRACLE trial): statistical analysis plan for a recursive two-stage group sequential randomized controlled trial. Trials 21(1), 8 (2020). 31900204 10.1186/s13063-019-3846-x PMC6942374

[29] Chu CM, Cheng VCC, Hung IFN Role of liponavir/ritonavir in the treatment of SARS: initial virological and clinical findings. Thorax 59(3), 252–256 (2004). 14985565 10.1136/thorax.2003.012658 PMC1746980

[30] Cao B, Wang Y, Wen D A trial of lopinavir-ritonavir in adults hospitalized with severe covid-19. N. Engl. J. Med. 382(19), 1787–1799 (2020). 32187464 10.1056/NEJMoa2001282 PMC7121492

[31] Li Y, Xie Z, Lin W Efficacy and safety of lopinavir/ritonavir or arbidol in adult patients with mild/moderate COVID-19: an exploratory randomized controlled trial. Med (NY) (2020) (Epub ahead of print). 10.1016/j.medj.2020.04.001 PMC7235585 32838353

[32] Cai Q, Yang M, Liu D Experimental treatment with favipiravir for COVID-19: an open-label control study. Engineering (Beijing) (2020) (Epub ahead of print). 10.1016/j.eng.2020.03.007 PMC7185795 32346491

[33] Clinical Trials Arena. Glenmark begins Phase III trials of favipiravir for COVID-19 in India. http://www.clinicaltrialsarena.com/news/glenmark-favipiravir-trial-begins/

[34] Grein J, Ohmagari N, Shin D Compassionate use of remdesivir for patients with COVID-19. N. Engl. J. Med. 382(24), 2327–2336 (2020). 32275812 10.1056/NEJMoa2007016 PMC7169476

[35] Wang Y, Zhang D, Du G Remdesivir in adults with severe COVID-19: a randomised double-blind, placebo-controlled, multicentre trial. Lancet 395(10236), 1569–1578 (2020). 32423584 10.1016/S0140-6736(20)31022-9 PMC7190303

[36] Beigel JH, Tomashek LE, Dodd AK Remdesivir for the treatment of COVID-19 – preliminary report. N. Engl. J. Med. (2020) (Epub ahead of print). 10.1056/NEJMc2022236 32649078

[37] Gautret P, Lagier J-C, Parola P Hydroxychloroquine and azothromycin as a treatment of COVID-19: results of an open-label non-randomized clinical trial. Int. J. Antimicrob. Agents 56(1), 105949 (2020). 32205204 10.1016/j.ijantimicag.2020.105949 PMC7102549

[38] Tang W, Cao Z, Han M Hydroxychloroquine in patients with mainly mild to moderate coronavirus disease 2019: open label, randomised controlled trial. Br. J. Med. 369, m1849 (2020). 10.1136/bmj.m1849 PMC7221473 32409561

[39] Ramsey ML, Nuttall J, Hart PA A Phase I/II trial to evaluate the pharmacokinetics, safety and efficacy of NI-03 in patients with chronic pancreatitis: study protocol for a randomized controlled trial on the assessment of camostat treatment in chronic pancreatitis (TACTIC). Trials 20(1), 501 (2019). 31412955 10.1186/s13063-019-3606-y PMC6694471

[40] Zhou Y, Vedantham P, Lu K Protease inhibitors targeting coronavirus and filovirus entry. Antiviral Res. 116, 76–84 (2015). 25666761 10.1016/j.antiviral.2015.01.011 PMC4774534

[41] Ota S, Hara Y, Kanoh S Acute eosinophilic pneumonia caused by camostat mesilate: the first case report. Respir. Med. Case Rep. 19, 21–23 (2016). 27408783 10.1016/j.rmcr.2016.06.005 PMC4925915

[42] Sayad B, Sobhani M, Khodarahmi R Sofosbuvir as repurposed antiviral drug against COVID-19: why were we convinced to evaluate the drug in a registered/approved clinicsal trial? Arch. Med. Res. S0188–4409(20), 30551–30558 (2020). 10.1016/j.arcmed.2020.04.018 PMC7188631 32387040

[43] Moghaddam E, Teoh BT, Sam SS Baicalin, a metabolite of baicalein with antiviral activity against dengue virus. Sci. Rep. 4, 5452 (2014). 24965553 10.1038/srep05452 PMC4071309

[44] Ho TY, Wu SL, Chen JC Emodin blocks the SARS coronavirus spike protein and angiotensin-converting enzyme 2 interaction. Antiviral Res. 74(2), 92–101 (2007). 16730806 10.1016/j.antiviral.2006.04.014 PMC7114332

[45] Marinella MA Indomethacin and resveratrol as potential treatment adjuncts for SARS-CoV-2/COVID-19. Int. J. Clin. Pract. 74(9), e13535 (2020). 32412158 10.1111/ijcp.13535 PMC7261995

[46] Zhang L, Lin D, Sun X Crystal structure of SARS-CoV-2 main protease provides a basis for design of improved αketoamide inhibitors. Science 368(6489), 409–412 (2020). 32198291 10.1126/science.abb3405 PMC7164518

[47] Kruse RL Therapeutic strategies in an outbreak scenario to treat the novel coronavirus originating in Wuhan, China. F1000Res 9, 72 (2020). 32117569 10.12688/f1000research.22211.2 PMC7029759

[48] Wong SK, Li W, Moore MJ A 193-amino acid fragment of the SARS coronavirus S protein efficiently binds angiotensin-converting enzyme 2. J. Biol. Chem. 279(5), 3197–3201 (2004). 14670965 10.1074/jbc.C300520200 PMC7982343

[49] Walls AC, Park YJ, Tortorici MA Structure, function and antigenicity of the SARS-CoV-2 spike glycoprotein. Cell 181(2), 281–292 (2020). 32155444 10.1016/j.cell.2020.02.058 PMC7102599

[50] Tian X, Li C, Huang A Potent binding of novel 2019 coronavirus spike protein by a SARS coronavirus-specific human monoclonal antibody. Emerg. Mircobes Infect. 9(1), 382–385 (2020). 10.1080/22221751.2020.1729069 PMC7048180 32065055

[51] Moen MD, Wagstaff AJ Losartan: a review of its use in stroke risk reduction in patients with hypertension and left ventricular hypertrophy. Drugs 65(18), 2657–2674 (2005). 16392883 10.2165/00003495-200565180-00012

[52] Kanters S, Socias ME, Paton NI Comparative efficacy and safety of second-line antiretroviral therapy for treatment of HIV/AIDS: a systematic review and network meta-analysis. Lancet HIV 4(10), e433–e441 (2017). 28784426 10.1016/S2352-3018(17)30109-1

[53] Osborne V, Davies M, Lane S Lopinavir-ritonavir in the treatment of COVID-19: a dynamic systematic benefit-risk assessment. Drug Saf. 43(8), 809–821 (2020). 32578156 10.1007/s40264-020-00966-9 PMC7309686

[54] Furuta Y, Komeno T, Nakamura T Favipiravir (T-705), a broad spectrum inhibitor of viral RNA polymerase. Proc. Jpn Acad. Ser. B Phys. Biol. Sci. 93(7), 449–463 (2017). 10.2183/pjab.93.027 PMC5713175 28769016

[55] Tripathy S, Dassarma B, Roy S A review on possible modes of chloroquine/hydroxychloroquine: repurposing against SARS-CoV-2 (COVID-19) pandemic. Int. J. Antimicrob. Agents 56(2), 106028 (2020). 32450198 10.1016/j.ijantimicag.2020.106028 PMC7243790

[56] Silva Borba MG, de Almeida Val F, Sousa Sampaio V Effect of high vs low doses of chloroquine diphosphate as adjunctive therapy for patients hospitalized with Severe Acute Respiratory Syndrome Coronavirus 2 (SARS-CoV-2) infection: a randomized clinical trial. JAMA Netw. Open 3(4), e208857 (2020). 32330277 10.1001/jamanetworkopen.2020.8857 PMC12124691

[57] Chorin E, Dai M, Shulman E The QT interval in patients with COVID-19 treated with hydroxychloroquine/azithromycin. Nat. Med. 26(6), 808–809 (2020). 32488217 10.1038/s41591-020-0888-2

[58] Uno Y Camostat mesylate therapy for COVID-19. Intern. Emerg. Med. (2020) (Epub ahead of print). 10.1007/s11739-020-02345-9 PMC7188520 32347443

[59] Hoffmann M, Kleine-Weber H, Schroeder S SARS-CoV-2 cell entry depends on ACE2 and TMPRSS2 and is blocked by a clinically proven protease inhibitor. Cell 181(2), 1–10 (2020). 32142651 10.1016/j.cell.2020.02.052 PMC7102627

[60] Camostat mesylate in COVID-19 outpatients. NCT04353284 (2020). https://clinicaltrials.gov/ct2/show/NCT04353284

[61] Buonaguro L, Buonagura F Knowledge-based repositioning of the anti-HCV direct antiviral agent sofosbuvir as SARS-CoV-2 treatment. Infect. Agent Cancer 15, 32 (2020). 32419838 10.1186/s13027-020-00302-x PMC7215134

[62] McKee DL, Sternberg A, Stange U Candidate drugs against SARS-CoV-2 and COVID-19. Pharmacol. Res. 157, 104859 (2020). 32360480 10.1016/j.phrs.2020.104859 PMC7189851

[63] Dai W, Bi J, Li F Antiviral efficacy of flavonoids against enterovirus 71 infection *in vitro* and in newborn mice. Viruses 11(7), 625 (2019). 10.3390/v11070625 PMC6669683 31284698

[64] Lin SC, Ho CT, Chuo WH Effective inhibition of MERS-CoV infection by resveratrol. BMC Infect. Dis. 17(1), 144 (2017). 28193191 10.1186/s12879-017-2253-8 PMC5307780

[65] Jin Z, Du X, Xu Y Structure of M(pro) from SARS-CoV-2 and discovery of its inhibitors. Nature 582(7811), 289–293 (2020). 32272481 10.1038/s41586-020-2223-y

[66] Du L, Kou Z, Ma C A truncated receptor-binding domain of MERS-CoV spike protein potently inhibits MERS-CoV infection and induces strong neutralizing antibody responses: implication for developing therapeutics and vaccines. PLoS ONE 8(12), e81587 (2013). 24324708 10.1371/journal.pone.0081587 PMC3852489

[67] Wang X, Mathieu M, Brezski RJ IgG Fc engineering to modulate antibody effector functions. Protein Cell 9(1), 63–73 (2018). 28986820 10.1007/s13238-017-0473-8 PMC5777978

[68] Li W, Moore MJ, Vasilieva N Angiotensin-converting enzyme 2 is a functional receptor for the SARS coronavirus. Nature 426(6965), 450–454 (2003). 14647384 10.1038/nature02145 PMC7095016

[69] Desmyter A, Farenc C, Mahony J Viral infection modulation and neutralization by camelid nanobodies. Proc. Natl Acad. Sci. USA 110(15), E1371–1379 (2013). 23530214 10.1073/pnas.1301336110 PMC3625315

[70] Koch K, Kalusche S, Torres JL Selection of nanobodies with broad neutralizing potential against primary HIV-1 strains using soluble subtype C gp140 envelope trimers. Sci. Rep. 7(1), 8390–8315 (2017). 28827559 10.1038/s41598-017-08273-7 PMC5566552

[71] Wang C, Li W, Drabek D A human monoclonal antibody blocking SARS-CoV-2 infection. Nat. Commun. 11(1), 2251 (2020). 32366817 10.1038/s41467-020-16256-y PMC7198537

[72] Gurwitz D Angiotensin receptor blockers as tentative SARS-CoV-2 therapeutics. Drug Dev. Res. 81(5), 537–540 (2020). 32129518 10.1002/ddr.21656 PMC7228359

[73] Lee RC, Feinbaum RL, Ambros V The *C. elegans* heterochronic gene lin-4 encodes small RNAs with antisense complimentary to lin-14. Cell 75(5), 843–854 (1993). 8252621 10.1016/0092-8674(93)90529-y

[74] Lundstrom K Micro-RNA in disease and gene therapy. Curr. Drug Discov. Technol. 8(2), 76–86 (2011). 21513487 10.2174/157016311795563857

[75] Shi Y Mammalian RNAi for the masses. Trends Genet. 19(1), 9–12 (2003). 12493242 10.1016/s0168-9525(02)00005-7

[76] Zhang Y, Li T, Fu L Silencing SARS-CoV Spike protein expression in cultured cells by RNA interference. FEBS Lett. 560(1–3), 141–146 (2004). 14988013 10.1016/S0014-5793(04)00087-0 PMC7127813

[77] Zheng BJ, Guan Y, Tang Q Prophylactic and therapeutic effects of small interfering RNA targeting SARS-coronavirus. Antivir. Res. 9(3), 365–374 (2004). 15259899

[78] Chang Z, Hu J RNAi therapeutics: Can siRNA conquer SARS? Gene Ther. 13(12), 871–872 (2006). 17262907 10.1038/sj.gt.3302682 PMC7091734

[79] Millet JK, Nal B Investigation of the functional roles of host cell proteins involved in coronavirus infection using highly specific and scalable RNA interference (RNAi) approach. Methods Mol. Biol. 1282, 231–240 (2015). 25720484 10.1007/978-1-4939-2438-7_19 PMC7121302

[80] Lu CY, Huang H-Y, Yang TH siRNA silencing of angiotensin-converting enzyme 2 reduced severe acute respiratory syndrome-associated coronavirus replications in Vero cells. Eur. J. Clin. Microbiol. Infect. Dis. 27(8), 709–715 (2008). 18449585 10.1007/s10096-008-0495-5 PMC7088151

[81] Nur SM, Hasan MA, Amin MA Design of Potential RNAi (miRNA and siRNA) Molecules for Middle East Respiratory Syndrome Coronavirus (MERS-CoV) Gene Silencing by Computational Method. Interdiscip. Sci. 7, 257–265 (2015). 26223545 10.1007/s12539-015-0266-9 PMC7090891

[82] Gu WY, Li Y, Liu BJ Short hairpin RNAs targeting M and N genes reduce replication of porcine deltacoronavirus in ST cells. Virus Genes 55(6), 795–801 (2019). 31463771 10.1007/s11262-019-01701-y PMC7088929

[83] Chen W, Feng P, Liu K Computational identification of small RNA targets in SARS-CoV-2. Virologica Sinica 35(3), 359–361 (2020). 32297156 10.1007/s12250-020-00221-6 PMC7157830

[84] Zhao WM, Song SH, Chen ML The 2019 novel coronavirus resource. Yi Chuan 42(2), 212–222 (2020). 32102777 10.16288/j.yczz.20-030

[85] WHO. DRAFT landscape of COVID-19 candidate vaccines. (2020). http://www.who.int/blueprint/priority-diseases/key-action/novel-coronavirus-landscape-ncov.pdf

[86] Thanh Le TT, Andreadakis Z, Kumar A The COVID-19 vaccine development landscape. Nat. Rev. Drug Discov. 19(5), 305–306 (2020). 32273591 10.1038/d41573-020-00073-5

[87] Codagenix and serum institute of india initiate co-development of a scalable, live-attenuated vaccine against the 2019 novel coronavirus, COVID-19. La Merie Publishing, (2020). https://pipelinereview.com

[88] Wang H, Zhang Y, Huang B Development of an inactivated vaccine candidate, BBIBP-CorV, with potent protection against SARS-CoV-2. Cell 182(3), 713–721 (2020). 32778225 10.1016/j.cell.2020.06.008 PMC7275151

[89] The University of Queensland, CEPI and CSL partner to advance development and manufacture of COVID-19 vaccine candidate. https://www.csl.com (2020).

[90] Chen W-H, Strych U, Hotez P The SARS-CoV-2 pipeline: an overview. Curr. Trop. Med. Rep. (2020) (Epub ahead of print). 10.1007/s40475-020-00201-6 PMC7094941 32219057

[91] Sanofi and GSK to join forces in unprecedented vaccine collaboration to fight COVID-19. https://ww.sanofi.com (2020).

[92] Van Doremalen N, Lambe T, Spencer A ChAdOx1 nCov-19 vaccination prevents SARS-CoV-2 pneumonia in rhesus macaques. Nature (2020) (Epub ahead of print). 10.1038/s41586-020-03099-2 33469217

[93] Li K, Li Z, Wohlford-Lenane C Single-dose, intranasal immunization with recombinant parainfluenza virus 5 expressing middle east respiratory syndrome coronavirus (MERS-CoV) spike protein protects mice from fatal MERS-CoV infection. mBio 11(2), e00554–20 (2020). 32265331 10.1128/mBio.00554-20 PMC7157776

[94] Stefano ML, Kream RM, Stefano GB A novel vaccine employing non-replicating rabies virus expressing chimeric SARS-CoV-2 spike protein domains: functional inhibition of viral/nicotinic acetylcholine receptor complexes. Med. Sci. Monit. 26, e926016 (2020). 32463026 10.12659/MSM.926016 PMC7278327

[95] COVID-19 vaccine development: an interview with GeoVax (2020). http://www.technologynetworks.com

[96] Themis collaborates with ABL Europe to manufacture its SARS-CoV-2 vaccine candidate in France (2020). http://www.themisbio.com

[97] Smith TRF, Patel A, Ramos S Immunogenicity of a DNA vaccine candidate for COVID-19. Nat. Commun. 11(1), 2601 (2020). 32433465 10.1038/s41467-020-16505-0 PMC7239918

[98] Yu J, Tostanoski LH, Peter L DNA vaccine protection against SARS-CoV-2 in rhesus macaques. Science 369(6505), 806–811 (2020). 32434945 10.1126/science.abc6284 PMC7243363

[99] About CureVac's activities regarding an mRNA-based vaccine against COVID-19 (2020). http://www.curevac.com/covid-19

[100] RNA manufacturing platform (2020). http://www.imperial.ac.uk/future-vaccine-hub/workstreams/rna-vaccine-manufacture/

[101] Sliepen K, van Montfort T, Melchers M Immunosilencing a highly immunogenic protein trimerization domain. J. Biol. Chem. 290(12), 7436–7432 (2015). 25635058 10.1074/jbc.M114.620534 PMC4367253

[102] Nikitin NA, Matveeva IN, Trifonova EA Spherical particles derived from TMV virions enhance the protective properties of the rabies vaccine. J. Data Brief 21, 742–745 (2018). 10.1016/j.dib.2018.10.030 PMC6214837 30406165

[103] Zhou Z, Post P, Chubet R A recombinant baculovirus-expressed S glycoprotein vaccine elicits high titers of SARS-associated coronavirus (SARS-CoV) neutralizing antibodies in mice. Vaccine 24(17), 3624–3631 (2006). 16497416 10.1016/j.vaccine.2006.01.059 PMC7115485

[104] Domi A, Feldmann F, Basu R A single dose of modified vaccinia ankara expressing ebola virus like particles protects nonhuman primates from lethal ebola virus challenge. Sci. Rep. 8(1), 864 (2018). 29339750 10.1038/s41598-017-19041-y PMC5770434

[105] Salvato MS, Domi A, Guzman-Cardazo a single dose of modified vaccinia ankara expressing lassa virus-like particles protects mice from lethal intra-cerebral virus challenge. Pathogens 8(3), 133 (2018). 10.3390/pathogens8030133 PMC6789566 31466243

[106] Brandler S, Ruffié C, Combridet C A recombinant measles vaccine expressing chikungunya virus-like particles is strongly immunogenic and protects mice from lethal challenge with chikungunya virus. Vaccine 31(36), 3718–3725 (2013). 23742993 10.1016/j.vaccine.2013.05.086

[107] Reisinger EC, Tschismarov R, Beubler E Immunogenicity, safety and tolerability of the measles-vectored chikungunya virus vaccine MV-CHIK: a double-blind, randomised, placebo-controlled and active-controlled Phase II trial. Lancet 392(10165), 2718–2727 (2019). 30409443 10.1016/S0140-6736(18)32488-7

[108] Lundstrom K Self-replicating RNA viruses for RNA therapeutics. Molecules 23(12), 3310 (2018). 10.3390/molecules23123310 PMC6321401 30551668

[109] Kose N, Fox JM, Sapparapu G A lipid-encapsulated mRNA encoding a potently neutralizing human monoclonal antibody protects against chikungunya infection. Sci. Immunol. 4(35), eaaw6647 (2019). 31101672 10.1126/sciimmunol.aaw6647 PMC6629435

[110] Wold WS, Toth K Adenovirus vectors for gene therapy, vaccination and cancer gene therapy. Curr. Gene 13(6), 421–433 (2013). 10.2174/1566523213666131125095046 PMC4507798 24279313

[111] A randomized, double-blind, placebo parallel-controlled Phase I/II clinical trial for inactivated Novel Coronavirus Pneumonia vaccine (Vero cells). ChiCTR2000031809. http://www.chictr.org.cn/showprojen.aspx?proj=52227

[112] A Phase I/II clinical trial for inactivated novel coronavirus (2019-CoV) vaccine (Vero cells). ChiCTR2000032459. http://www.chictr.org.cn/showproj.aspx?proj=53003

[113] China steps up inactivated COVID-19 vaccine development. http://www.xinhuanet.com/english/2020-06/01/c_139105909.htm (2020).

[114] Gao Q, Bao L, Mao H Development of an inactivated vaccine candidate for SARS-CoV-2. Science 369(6499), 77–81 (2020). 32376603 10.1126/science.abc1932 PMC7202686

[115] Safety and immunogenicity study of inactivated vaccine for prevention of SARS-CoV-2 infection (COVID-19). NCT04383574. https://clinicaltrials.gov/ct2/show/NCT04383574

[116] Safety and immunogenicity study of inactivated vaccine for prophylaxis of SARS CoV-2 infection (COVID-19). NCT04352608. https://clinicaltrials.gov/ct2/show/NCT04352608?term=Sinovac&cntry=CN&draw=2

[117] The updated list of COVID-19 vaccines (2020). https://covidvax.org

[118] Lövgren Bengtsson K, Song H, Stertman L Matrix-M adjuvant enhances antibody, cellular and protective immune responses of a Zaire Ebola/Makona virus glycoprotein (GP) nanoparticle vaccine. Vaccine 34(16), 1927–1935 (2016). 26921779 10.1016/j.vaccine.2016.02.033

[119] Novavax initiates Phase I/II clinical trial of COVID-19 vaccine (2020). https://ir.novavax.com

[120] Evaluation of the safety and immunogenicity of a SARS-CoV-2 rS (COVID-19) nanoparticle vaccine with/without matrix-M adjuvant. NCT04368988. https://clinicaltrials.gov/ct2/show/NCT04368988?term=vaccine&recrs=a&cond=covid-19&draw=2

[121] A Phase I/II study to determine efficacy, safety and immunogenicity of the candidate coronavirus disease (COVID-19) vaccine ChAdOx1 nCoV-19 in UK healthy adult volunteers. 2020-001072-15. http://www.clinicaltrialsregister.eu/ctr-search/trial/2020-001072-15/GB

[122] Recruitment to this Phase I/II study (COV001) is now complete. Recruitment to the Phase II/III study is ongoing (2020). https://covid19vaccinetrial.co.uk/ongoing-studies

[123] A Phase II/III study to determine the efficacy, safety and immunogenicity of the candidate coronavirus disease (COVID-19) vaccine ChAdOx1 nCoV-19. 2020-001228-32. http://www.clinicaltrialsregister.eu/ctr-search/trial/2020-001228-32/GB

[124] A Phase I clinical trial for recombinant novel coronavirus (2019-COV) vaccine (adenoviral vector) ChiCTR2000030906. http://www.chictr.org.cn/showprojen.aspx?proj=51154

[125] Zhu F-C, Li Y-H, Guan X-H Safety, tolerability and immunogenicity of a recombinant adenovirus type-5 vectored COVID-19 vaccine: a dose-escalation, open-label, non-randomised, first-in-human trial. Lancet 396(10249), 479–488 (2020). 32450106 10.1016/S0140-6736(20)31208-3 PMC7255193

[126] A randomized, double-blinded, placebo-controlled Phase II clinical trial for Recombinant Novel Coronavirus (2019-nCOV) Vaccine (Adenovirus Vector) in healthy adults aged above 18 years. ChiCTR2000031781. http://www.chictr.org.cn/showprojen.aspx?proj=52006

[127] China's Shenzhen Geno-Immune Medical Institute pursues a COVID-19 vaccine (2020). http://www.trialsitenews.com/category/shenzhen-geno-immune-medical-institute/

[128] Inovio urgently focused on developing COVID-19 vaccine (2020). http://www.inovio.com/our-focus-serving-patients/covid-19/

[129] Moderna advances late-stage development of its vaccine (mRNA-1273) against COVID-19 (2020). https://investors.modernatx.com/news-releases/news-release-details/moderna-advances-late-stage-development-its-vaccine-mrna-1273

[130] Safety and immunogenicity study of 2019-nCoV Vaccine (mRNA-1273) for prophylaxis of SARS-CoV-2 infection (COVID-19). NCT04283461. https://clinicaltrials.gov/ct2/show/NCT04283461?term=vaccine&cond=covid-19&draw=2

[131] Moderna's latest vaccine results are promising – but it's still too early (2020). http://www.technologyreview.com/2020/05/18/1001834/moderna-coronavirus-vaccine-phase-i-interim-clinical-trial-results/

[132] Study to describe the safety, tolerability, immunogenicity and efficacy of rna vaccine candidates against COVID-19 in healthy adults. NCT04368728. https://clinicaltrials.gov/ct2/show/NCT04368728?term=vaccine&cond=covid-19&draw=3

[133] Norton Td, Zhen A, Tada T Lentiviral vector-based dendritic cell vaccine suppresses HIV replication in humanized mice. Mol. Ther. 27(5), 960–973 (2019). 30962161 10.1016/j.ymthe.2019.03.008 PMC6520467

[134] Bloch EM, Shoham S, Casadevall A Deployment of convalescent plasma for the prevention and treatment of COVID-19. J. Clin. Investig. 130(6), 2757–2765 (2020). 32254064 10.1172/JCI138745 PMC7259988

[135] Rajendran K, Krishnasamy N, Rangarajan J Convalescent plasma transfusion for the treatment of COVID-19: systemic review. J. Med. Virol. (2020) (Epub ahead of print). 10.1002/jmv.25961 PMC7267113 32356910

[136] Zhang L, Pang R, Xue X Anti-SARS-CoV-2 virus antibody levels in convalescent plasma of six donors who have recovered from COVID-19. Aging 12(8), 6536–6542 (2020). 32320384 10.18632/aging.103102 PMC7202482

